# Magnetic compression anastomosis for rectal atresia following necrotizing enterocolitis: A case report

**DOI:** 10.1097/MD.0000000000023613

**Published:** 2020-12-11

**Authors:** Shi-Qi Liu, Qi-Feng Li, Yi Lv, Jing-Ru Zhao, Rui-Xue Luo, Peng-Fei Zhang, Jin-Zhen Guo, An-Peng Zhang, Qing-Hong Li

**Affiliations:** a Department of Neonatal Surgery, the Children Hospital of Xi’an City, Xi’an; b Xinjiang Institute of Pediatrics, People's Hospital of Xinjiang Uygur Autonomous Region, Urumqi, Xinjiang Uygur Autonomous Region; c Department of Hepatobiliary Surgery, The First Affiliated Hospital of Xi’an Jiaotong University; d Department of Pediatrics, The Northwest Women's and Children's Hospital; e Corrosion & Protection Research Lab, The Northwest Institute for Nonferrous Metal Research (NIN); f Department of Neonatal Intensive Care Unit, The Northwest Women's and Children's Hospital, Xi’an, Shanxi, China.

**Keywords:** children, magnamosis, magnetic compression anastomosis, necrotizing enterocolitis, rectal atresia

## Abstract

**Rationale:**

Rectal atresia caused by necrotizing enterocolitis (NEC) is a serious and rare complication in children. Magnetic compression anastomosis (MCA) has been effectively applied in children with congenital oesophageal atresia and biliary atresia. Herein, we reported a case of successfully application of MCA in an infant with rectal atresia following NEC.

**Patient concerns:**

A 30^+6^ weeks premature birth female fetal infant was transferred to our neonatal intensive care unit due to premature delivery, low birth weight, and neonatal respiratory distress. On postpartum day 11, the infant developed abdominal distension and mucosanguineous feces. This infant was then clinically diagnosed as NEC. She underwent anesthesia and intestinal fistula operation on postpartum day 11 because of NEC.

**Diagnosis:**

After 3 months, radiographic examination revealed rectal atresia and stricture.

**Interventions:**

This infant was successfully treated with MCA following a cecum-rectal anastomosis and ileocecal valve was reserved.

**Outcomes:**

On postoperative day 9, she passed the 2 magnets per rectum. In addition, there were no difficult defecation or fecal incontinence or other short-term complications. After the 7-month follow-up, the patient had an excellent clinical outcome.

**Lessons:**

MCA is a feasible and effective method for treating rectal atresia in infants.

## 1 Introduction

Necrotizing enterocolitis (NEC) is a common gastrointestinal inflammatory disease in premature infants.^[1]^ Rectal atresia is a serious and rare complication following NEC^[2]^ and needs transanal anastomosis. It is difficult for a low rectal anastomosis by manual suture for children. Therefore, the surgical treatment for rectal atresia has been considered as a significant challenge for pediatric surgeons.

Magnetic compression anastomosis (MCA) is a sutureless serosa-to-serosa anastomosis for the gastrointestinal tract and is created by the compression of 2 magnets (parent and daughter) placing on each side of the tract wall, which has been effectively applied in children with congenital oesophageal atresia^[2]^ and biliary atresia.^[3]^ To our knowledge, there is no report of MCA in children with rectal atresia following NEC. In the present case report, we described a case of infant with rectal atresia following NEC who received colorectal anastomosis by MCA.

## 2 Case presentation

**Figure 1:**
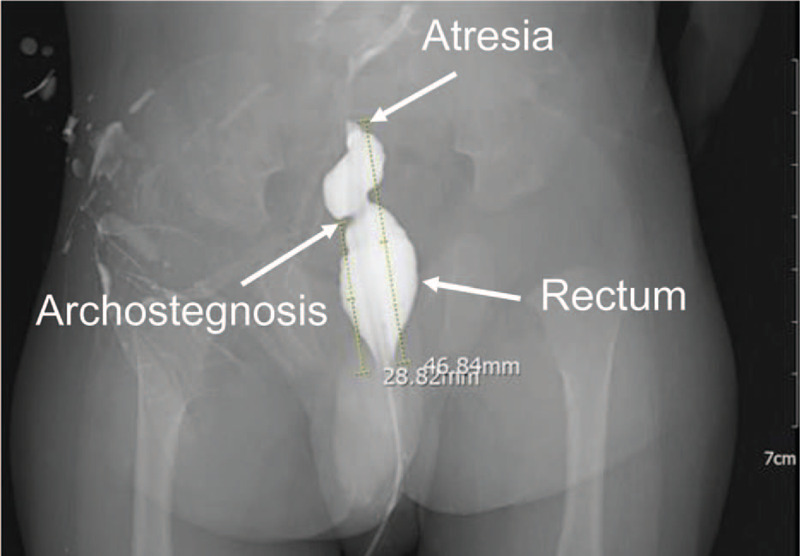
Preoperative anteroposterior contrast enema showing rectal atresia and the distal blind end of the rectal lies at the level of 3 cm from the anal margin.

A female fetal with a 1660 g weight was born at 30^+6^ weeks following in vitro fertilization - embryo transplantation and twin pregnancy, and was transferred to our neonatal intensive care unit due to premature delivery, low birth weight, and neonatal respiratory distress. According to results of the blood test and ultrasound, this infant had no cardiac, spinal, urinary or other congenital malformations. The anal opening was located within the center of a normal sphincter complex. On postpartum day 11, the infant developed abdominal distension and mucosanguineous feces. An emergency plain abdominal X-ray revealed dilated loops of bowel with pneumatosis intestinalis and hepatic portal venous gas. This infant was then clinically diagnosed as NEC. Therefore, intestinal fistula was performed at 15 cm distal from the ileocecal region. After 3 months, radiographic examination revealed rectal atresia and stricture (Fig. 1).

**Figure 2:**
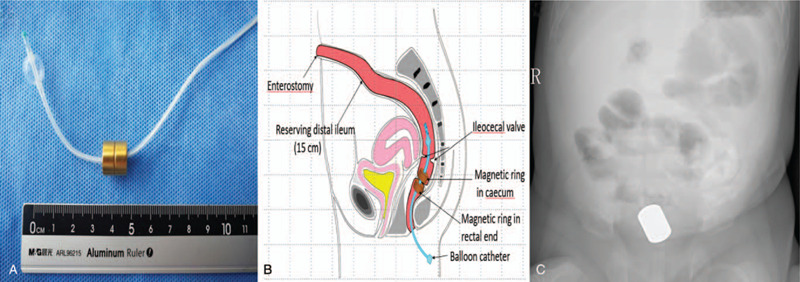
Design and placement of the magnetic rings. (A) The magnetic equipment designed and used for the present case. The matched ring had a same outer diameter of 12 mm with thickness of 6 mm. (B) The schematic presentation of the axially magnet rings to be placed into the upper and distal ends of the bowel lumen, respectively. The mother ring was scheduled to be placed in the proximal end of the caecum, while the daughter ring was planned to be positioned in distal rectum end. A balloon catheter was to be placed through the central hole of both rings and the bowel blind pouch, reaching the intestinal cavity. The contact of the 2 bowel ends was anticipated to achieve cecum-rectal anastomosis. (C) Opposed magnets following passage of 1 magnet through the mucous fistula to the pouch suture proximal caecum and one per rectum to the blind end of rectum along an 8F soft balloon catheter, respectively. The contact of the 2 intestine ends was anticipated to achieve anastomosis.

Two magnetic rings, 1 mother ring, and 1 daughter ring, with a strength of 2,500 G force and low noxious property (Fig. 2A), were customized with a suction power between the cecum and distal rectal pouches (Fig. 2B). Briefly, the MCA was performed during the second stage surgery of NEC. One magnet ring (mother ring) with an outer diameter of 12 mm and a thickness of 6 mm was placed in the proximal cecum pouch, while the second magnet ring (daughter ring) with an outer diameter of 12 mm and a thickness of 6 mm was positioned in the distal rectal lumen. Subsequently, the 8F balloon catheter went through the central hole of the mother ring and the daughter ring. Under the effect of the magnetic force, the 2 magnet rings pulled along the catheter to achieve cecum-rectal anastomosis.

By using magnet therapy, any disruption to the sphincter mechanism and associated nerves may be avoided. After several communications with her parents, it was decided in agreement to attempt to approximate the 2 pouches by magnet force, as previously reported.^[4]^ At age of 4 months, the patient underwent rectocecal anastomosis, and the distance has been confirmed less than 3 cm from blind end to anal edge in the rectum. A 12 mm magnet ring through the mucous fistula and a second magnet through the anus was implanted respectively to create a magnamosis between the distal and proximal pouches (Fig. 2C).

The permanent suction of these 2 pouches was established following surgery. Enteral nutrition was administered via transoral boluses. Antibiotics were administered, and feeding via oral was performed after the operation. There's no increasing in leucocyte count in peripheral blood. The postoperative C-reactive protein level was 35 mg/L, which normalized within 3 days.

**Figure 3:**
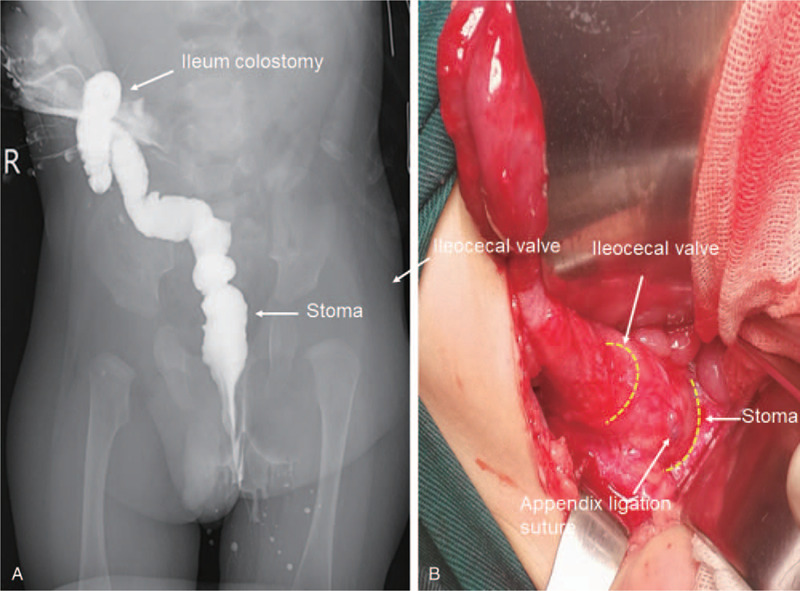
Serial post-magnamosis radiographs of X-ray images and surgical findings. (A) Post-magnamosis contrast enema demonstrating the absence of stricture; (B) Surgical for fistula closing reveals the anastomotic site soft and healing well without stricture (the dotted line is the location of the anastomosis).

The magnetic coupling was observed during the operation. And anastomotic healing was achieved finally. The magnetic rings were expelled naturally through the anus at day 9 post-operation without perforations or other early complications. After 3 months, a barium enema demonstrated an intact anastomosis without stricture (Fig. 3A). At 7 months after birth, this patient received fistula closure operation. During operation we checked the caecorectal stoma and confirmed no stenosis (Fig. 3B). Thereafter, the parents performed daily rectal dilations to prevent anastomotic stenosis. Furthermore, body-weight and height increased normally. At age of 7 months, she successfully underwent surgery to close colostomy and to restore gastrointestinal continuity. Until the completion of this manuscript, this patient is 1 year old with normal bowel function. Furthermore, her psychomotor development, height, and weight are presented as normal, according to the standard curve for Chinese children.

## 3 Discussion

In most instances, the colorectal stricture is clinically manifested around 5 to 7 weeks after the onset of NEC and the majority of strictures after NEC is located in colon region.^[1]^ Rectal atresia caused by NEC is a serious and rare complication that requires staged operative management, which still is considered as a challenging for pediatric surgeons. Several operative techniques including various pull-through procedures, transanal rectorectal anastomosis,^[5]^ posterior sagittal repair,^[6]^ and endoscopic techniques^[7]^ have been described for management of this condition, but there is no clear consensus on best management yet.

In the present case, the female patient was confirmed as rectal atresia after NEC. The distance from blind end to anal edge in the rectum was approximately 3 cm. And there was no effective method for the significant spontaneous growth and elongation of the pouches.

According to the experience of the surgeon, in setting of available optimal preoperative imaging, this patient's rectal atresia after NEC may be definitively repaired at age of several months. Different from congenital anorectal malformations, this NEC patient suffered from rectal atresia but had favorable anorectum anatomy. Magnamosis offers a minimally invasive technique as an option for rectal atresia repair anomaly, which may serve as an alternative option for operative repair such as posterior sagittal reconstruction. Further investigation and refinement of magnamosis is required before it can be recommended in clinical practice.

Multiple reports have described intestinal fistulas resulting from accidental magnet ingestion.^[8–11]^ This observation introduced the idea that rectal atresia could be treated with an anastomosis formed by rare earth magnets (magnamosis).^[4]^ Magnamosis has been successfully used in treatment of benign and malignant biliary strictures, magnetic connectors for coronary surgery,^[12,13]^ and the functional magnamosis undiversion procedure of ileostomy in pediatric patients.^[14]^ At present, MCA for the treatment of esophageal atresia patients is restricted to gross type A (without tracheoesophageal fistula^[15]^ and anastomotic stenosis after definitive esophago-esophagostomy without thoracotomy.^[16,17]^ To the best of our knowledge, the present study is the first report presenting a rare case of rectal atresia caused by neonatal NEC, and reported the feasibility and efficacy of MCA in achieving anastomosis. The successful treatment of rectal atresia in the infant may merit attention.

We propose that magnamosis can be considered as an alternative treatment option for rectal atresia in certain children. Anastomotic stricture is a primary concern when using this technique. We recommend scheduled anastomotic dilations per rectum similar to those performed in children with other anorectal malformations repaired using the traditional posterior sagittal anorectoplasty. For the magnamosis, the shape and strength of magnets were adapted to the rectal diameter. Further design refinements are necessary, especially to reduce the incidence of postanastomotic stenosis.

It was suggested that the duration to develop a solid anastomosis and the time point for the easy removal of the magnets should be ranged within 2 weeks following surgery.^[18]^ In this case, the anastomosis was achieved on day 9 after surgery. Since the strength of the magnets affect the duration to develop a solid anastomosis, the duration to develop a solid anastomosis can be shortened if the field strength of the magnetic rings is increased. The mother and daughter rings should produce a magnetic compression power lower than 12,000 G.^[17,19]^

Importantly, the attractive force between magnets exponentially increases as the distance of separation decreases. Due to this property of magnetism, there is a risk of intestinal tract tearing or perforation resulted from the excessive force, as the intestinal tissue decreases during treatment.

This inverse exponential behavior of force produces an increased rate of compression that is highly different from the ideal gradual stretch, growth and compression desired to achieve the anastomosis. In the present case, it was found that the magnetic coupling occurred more rapidly than anticipated. No severe short-term complications were observed.

In summary, the successful anastomosis with magnetic compression in the present case with rectal atresia after NEC is worthy of attention. These findings suggest that MCA is a feasible and effective nonsurgical treatment for rectal atresia in infants.

## Acknowledgments

The authors thank the Northwest Institute for Nonferrous Metal Research (NIN) for the Nv-Fe-P material processing and magnetic rings production. We are also grateful to the staff of the Medical Record Information Management Department for their support and assistance.

## Author contributions

Yi Lv and Jing-Ru Zhao guided the operation and revised the manuscript. Shi-Qi Liu and Qi-Feng Li conducted the operation and drafted this manuscript. Rui-Xue Luo assisted in the magnetic rings processing. Peng-Fei Zhang and An-Peng Zhang provided assistance in the operation, and contributed to the perioperative management. Jin-Zhen Guo and Qing-Hong Li contributed to the NICU management. All authors have read and approved the final manuscript.
